# Nomograms for predicting specific distant metastatic sites and overall survival of colorectal cancer patients: A large population‐based real‐world study

**DOI:** 10.1002/ctm2.20

**Published:** 2020-04-29

**Authors:** Shaobo Mo, Xin Cai, Zheng Zhou, Yaqi Li, Xiang Hu, Xiaoji Ma, Long Zhang, Sanjun Cai, Junjie Peng

**Affiliations:** ^1^ Department of Colorectal Surgery Fudan University Shanghai Cancer Center Shanghai China; ^2^ Department of Oncology, Shanghai Medical College Fudan University Shanghai China; ^3^ Department of Radiation Oncology Shanghai Proton and Heavy Ion Center Shanghai China; ^4^ Shanghai Engineering Research Center of Proton and Heavy Ion Radiation Therapy Shanghai China; ^5^ Department of Cancer Institute Fudan University Shanghai Cancer Center Fudan University Shanghai China

**Keywords:** colorectal cancer, decision curve analysis, distant metastasis, nomogram, overall survival

## Abstract

**Background:**

This study aims to develop functional nomograms to predict specific distant metastatic sites and overall survival (OS) of colorectal cancer (CRC) patients.

**Methods:**

CRC case data were retrospectively recruited from a large population‐based public dataset. Nomograms were developed to predict the probabilities of specific distant metastatic sites and OS of CRC patients. The performance of nomogram was evaluated with the concordance index (C‐index), calibration curves, area under the curve (AUC), and decision curve analysis (DCA).

**Results:**

A total of 142 343 cases were included in the current study. On the basis of univariate and multivariate analyses, clinicopathological features were correlated with specific distant metastatic sites and survival outcomes and were used to establish nomograms. The nomograms showed excellent accuracy in predicting specific distant metastatic sites. The C‐indexes for the prediction of liver, lung, bone, and brain metastases were 0.82 (95% confidence interval (CI), 0.81‐0.83), 0.80 (95% CI, 0.78‐0.81), 0.83 (95% CI, 0.79‐0.86), and 0.73 (95% CI, 0.72‐0.84), respectively. Then, a prognostic nomogram integrating clinicopathological features and specific distant metastatic sites was established to predict 1‐, 3‐, and 5‐year OS of CRC, with AUCs of 0.764 (95% CI, 0.741‐0.783), 0.762 (95% CI, 0.745‐0.781), and 0.745 (95% CI, 0.730‐0.761), respectively. DCA showed that the prognostic nomogram had a better clinical application value than current TNM staging system.

**Conclusions:**

Based on clinicopathological features, original nomograms were constructed for clinicians to predict specific distant metastatic sites and OS of CRC patients. These models could help to support the postoperative personalized assessment.

**Abbreviations**
AD: adenocarcinoma
AUC: the area under the curve
CEA: carcinoembryonic antigen
CI: confidence interval
C‐index: The concordance index
CRC: colorectal cancer
DCA: decision curve analysis
dMMR: mismatch repair deficiency
HR: hazard ratio
IQR: interquartile range
LNH: lymph nodes harvested
MAD: mucinous adenocarcinoma
MSI: microsatellite instability
OS: overall survival
ROC: receiver operating characteristic
SD: standard deviation
SEER: Surveillance, Epidemiology, and End Results
SRCC: signet ring cell carcinoma
TNM: tumor‐node‐metastasis

## 1 BACKGROUND

In 2019, there were 148 000 new cases of colorectal cancer (CRC), which accounted for more than 146 deaths per day, with an approximately 19.1/100 000 mortality rate, ranking third among all malignant tumors in the United States.^1^ Over the past 30 years, the incidence and overall survival (OS) rate of CRC have been rising worldwide. The 5‐year OS rate of CRC patients was approximately 65.2%. An important reason for limited 5‐year survival in CRC patients is distant metastasis, including liver, lung, brain, and bone metastasis.

Current research has indicated that clinicopathological characteristics such as histological classification, pretreatment carcinoembryonic antigen (CEA) levels, distant metastasis site, and depth of infiltration may also affect survival outcomes in patients with CRC.^2^ The postoperative survival of CRC patients with different clinicopathological features varies greatly. For instance, the 5‐year survival rate of CRC patients with distant metastases, such as brain metastases, is less than 10%, while the 5‐year survival rate of CRC patients with infiltration not exceeding the muscular layer reaches more than 90%.^3^ The prognosis of CRC patients varies in different clinicopathological factors. Therefore, a statistical model tool is required to comprehensively combine the effect of various clinicopathological elements on the outcomes of CRC patients.

The prognosis of CRC is associated with the current tumor‐node‐metastasis (TNM) staging system. For patients with the same tumor stage, prognosis can significantly vary because of the heterogeneity of CRC. Although TNM staging system is extensively used in the postoperative decision‐making for treatment strategy and prognosis evaluation of CRC patients in current clinical practice, its existing shortcomings cannot be ignored, which has been widely studied in recent years.^4^ Accumulating prognostic biomarkers have been explored, studied and applied in clinical practice to make up for the deficiency of current TNM classification system. For example, microsatellite instability (MSI)/mismatch repair (MMR) status has been recommended to be the most commonly used and powerful molecular marker in the clinical management of CRC patients.^5^, ^6^ In addition, the expression statuses of diverse genes, such as KRAS and BRAF, have been found to be closely related to the prognosis of CRC patients.^7^, ^8^ Nevertheless, both immunohistochemistry and gene detection methods have limitations, but also bring a certain economic burden to patients. At present, researchers have been trying to develop and validate the nomogram to predict patients’ prognosis by using the clinicopathological data. Through the combination of clinicopathological features, the nomogram can clearly and intuitively quantify the local recurrence chances, distant metastasis rates, and survival probabilities of patients with cancer. These studies have also achieved corresponding success.^9^, ^10^ Currently, most nomogram models cannot predict all metastatic sites and probabilities of patients, or the metastatic states are not included when constructing these models, which limits their clinical application.

With the application of a large population‐based public dataset, the Surveillance, Epidemiology, and End Results (SEER) program that provides extensive clinicopathological and follow‐up information of cancer patients, covering about 28% of the population in the United States, researchers have carried out a large number of clinical studies on different cancers.^11^ In this study, all CRC cases with clinicopathological and survival information were collected from the SEER program to establish intuitive and comprehensive nomograms for predicting specific distant metastatic sites and prognosis in CRC patients.

## 2 METHODS

### 2.1 Patients

**FIGURE 1:**
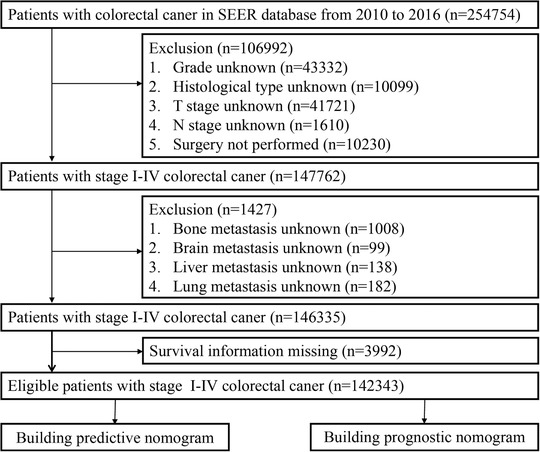
Recruitment pathway of CRC patients with specific metastasis sites and complete follow‐up information to establish predictive and prognostic nomograms

In present study, a total of 254 754 cases were obtained from the SEER cohort. The flow chart of case inclusion and exclusion is shown in Figure 1. All cases who received radical operation from 2010 to 2016 were involved in the study and analyzed retrospectively. Cases with no or multiple tumors in the pathological report were excluded. Accordingly, 15 clinicopathological characteristics were extracted from SEER program, including gender, race, tumor location, pathological grade, histological type, age at diagnosis, tumor size, pretreatment CEA level, number of lymph nodes harvested (LNH), T stage, N stage, specific distant metastatic sites (liver, lung, bone, and brain). If recorded as Unknown, Asian, Native American (NA), or Pacific Islander (PI), cases were allocated to an “other” race category for analysis. The exclusion criteria are as follows: (a) Absence of important clinicopathological factors, such as grade, histological type, T stage, and N stage. (b) Loss of specific metastatic sites (lung metastases, liver metastases, bone metastases, or brain metastases). (c) Incomplete survival information (survival months and survival status). Patient survival was measured by OS.^12^ Finally, 142 343 patients with stage I‐IV CRC, ensuring a full range of clinical and pathological data, were chosen from the SEER database.

### 2.2 Construction of prediction and prognostic nomograms

Univariate and multivariate logistic regression and Cox regression analyses identified independent prognostic factors for specific distant metastasis site and OS, respectively, and hazard ratio (HR) was used to measure the impact of each independent prognostic factor on specific distant metastasis site and OS, respectively. Then, according to the results of multivariate logistic regression analysis, predictive nomograms were established to predict the risk of liver, lung, bone, and brain metastasis in CRC patients. Meanwhile, combined with four specific distant metastasis sites, one prognostic nomogram was recommended to predict the OS probability of CRC patients. Based on the nomogram scoring, cases were classified as low‐, moderate‐, and high‐risk subgroups.

### 2.3 Receiver operating characteristic (ROC) curves and prediction error curves

The differentiation ability of the nomograms was evaluated by ROC and calibration curves. The accuracy in predicting distant metastases was measured by the logistic ROC and calibration curves. Application of ROC curves in 1‐, 3‐, and 5‐year survival probability to evaluate the prediction ability of nomogram over time. The value of AUC is the same as that of the concordance index (c‐index) in logistic regression model. The maximum AUC is 1.0, indicating a perfect discrimination, while 0.5 stands for a random chance to correctly identify the nomogram. The prediction error curve of the model was used to compare the TNM staging system error rate with that of the prognostic nomogram over time.^13^

### 2.4 Decision curve analysis

As a novel tool to evaluate the nomogram in clinical application value, decision curve analysis (DCA) was performed in present study as a method for assessing the predictive models’ ability to visualize the clinical outcomes, and was conducted to compare the net benefit of the predictive and prognostic nomograms.^14^ The aim of DCA is to evaluate the risk of adverse outcomes of individuals, and to suggest some intervention or treatment for sufficiently high‐risk individuals.

### 2.5 Risk stratification

In order to test the discrimination of nomogram, all cases were redefined as low‐, medium‐, and high‐risk subgroups based on the eventual risk score. The survival curves of different risk subgroups were drawn by Kaplan‐Meier method and evaluated by log‐rank test.

### 2.6 Statistical analysis

All statistical analyses were performed by R software (http://www.r-project.org, version 3.3.3). In the present study, the following R packages were downloaded to build nomogram, plot ROC curves, calibration, and DCA curve, and to draw the Kaplan‐Meier curves: “Hmisc,” “survival,” “rms,” “pROC,” “survivalROC,” “MASS,” and “rmda.” All statistical tests were two‐sided, with *P*‐values < .05 considered statistically significant.

## 3 RESULTS

### 3.1 Patient characteristics and outcomes

A total of 142 343 stage I‐IV CRC patients were retrospectively enrolled from SEER database. The patients’ demographics and clinical features are listed in Table 1. The mean follow‐up period was 38 ± 22 months. In the whole group, the median age was 67 years old (interquartile range, 57.0‐77.0). Most patients (79.2%) were white, and more than half of the patients (51.9%) were male. Most cases were of the adenocarcinoma histological type (129 979; 91.3%) and grade II (104 155; 73.2%) and had LNH ≥12 (112 538; 79.1%). Moreover, more than half of the patients had tumor size <5 and were in the pT3 stage and pN0 stage. For the patients with available CEA information, more than half of the patients (58.3%) were CEA negative. For distant metastatic sites, tumors with liver, lung, bone, and brain metastases accounted for 8.1%, 1.9%, 0.3%, and 0.1% of all cases, respectively.

**TABLE 1:** Demographics and clinical characteristics of colorectal cancer patients

Characteristics	Level	Number (%)
Age at diagnosis	Mean ± SD	66.7 ± 13.7
	Median (IQR)	67.0 (57.0‐77.0)
	<60	46 873 (32.9)
	≥60	95 470 (67.1)
Race	White	112 754 (79.2)
	Black	16 109 (11.3)
	Other	13 480 (9.5)
Gender	Female	68 409 (48.1)
	Male	73 934 (51.9)
Site	Right colon	70 313 (49.4)
	Left colon	47 471 (33.3)
	Rectum	24 559 (17.3)
CEA	Positive	34 939 (24.5)
	Negative	48 655 (34.2)
	Other	58 749 (41.3)
Grade	I	12 227 (8.6)
	II	104 155 (73.2)
	III	21 649 (15.2)
	IV	4312 (3.0)
Histological type	AD	129 979 (91.3)
	MAD	11 031 (7.7)
	SRCC	1333 (1.0)
Tumor size	<5	78 519 (55.2)
	≥5	63 824 (44.8)
LNH	<12	29 805 (20.9)
	≥12	112 538 (79.1)
T stage	T1	23 087 (16.2)
	T2	21 813 (15.3)
	T3	75 209 (52.9)
	T4	22 234 (15.6)
N stage	N0	83 096 (58.4)
	N1	37 387 (26.3)
	N2	21 860 (15.3)
M stage	M0	125 732 (88.3)
	M1	16 611 (11.7)
pTNM	I	37 535 (26.4)
	II	42 501 (29.8)
	III	45 696 (32.1)
	IV	16 611 (11.7)
Liver metastasis	No	130 813 (91.9)
	Yes	11 530 (8.1)
Lung metastasis	No	139 595 (98.1)
	Yes	2748 (1.9)
Bone metastasis	No	141 931 (99.7)
	Yes	412 (0.3)
Brain metastasis	No	142 221 (99.9)
	Yes	122 (0.1)

Abbreviations: SD, standard deviation; IQR, interquartile range; CEA, carcinoembryonic antigen; AD, adenocarcinoma; MAD, mucinous adenocarcinoma; SRCC, signet‐ring cell carcinoma; LNH, lymph node harvested.

### 3.2 Construction of predictive nomograms for specific distant metastatic sites

**FIGURE 2:**
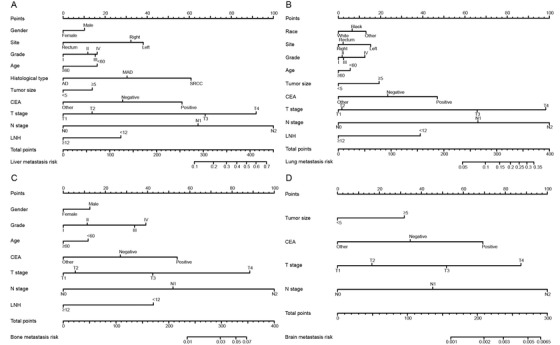
Four nomograms convey the results of predictive models using clinicopathological characteristics to predict the possibility of liver (**A**), lung (**B**), bone (**C**), and brain (**D**) metastasis

Univariate logistic regression for the presence of different metastatic sites showed that 11 variables, including race, sex, age, CEA level, grade, tumor site, histological type, tumor size, LNH, N stage, and T stage were related to distant metastatic sites (Table 2). In multivariate logistic regression (Table 3), the vast majority of variables, including sex, age, tumor site, CEA level, grade, histological type, tumor size, N stage, T stage, and LNH were determined as independent risk factors predicting liver metastases of CRC. Nine parameters, including race, age, tumor site, CEA level, grade, tumor size, N stage, LNH, and T stage, were determined to be independent risk factors predicting lung metastases of CRC. Seven comparable parameters, including sex, age, CEA level, grade, N stage, LNH, and T stage were identified as independent risk factors predicting bone metastases of CRC. Four factors, including CEA level, N stage, T stage, and tumor size were defined as independent risk factors predicting brain metastases of CRC. On the basis of multivariable logistic regression analyses for specific distant metastatic sites, all of the independent significant risk factors were integrated to establish nomograms for specific metastatic site prediction. The predictive nomograms for liver (Figure 2A), lung (Figure 2B), bone (Figure 2C), and brain (Figure 2D) metastases are illustrated in Figure 2.

**TABLE 2:** Univariate logistic regression for the presence of different metastatic sites at diagnosis of colorectal cancer

	Liver metastasis		Lung metastasis		Bone metastasis		Brain metastasis	
Characteristics	HR (95% CI)	*P*‐value	HR (95% CI)	*P*‐value	HR (95% CI)	*P*‐value	HR (95% CI)	*P*‐value
Age at diagnosis		<.001		<.001		<.001		.014
<60	1		1		1		1	
≥60	0.629 (0.605‐0.654)	<.001	0.724 (0.670‐0.782)	<.001	0.684 (0.562‐0.833)	<.001	0.639 (0.447‐0.914)	.014
Race		<.001		<.001		.076		.538
White	1		1		1		1	
Black	1.450 (1.374‐1.531)	<.001	1.283 (1.136‐1.449)	<.001	0.926 (0.653‐1.314)	.668	0.676 (0.329‐1.389)	.832
Other	0.987 (0.923‐1.055)	.701	1.540 (1.387‐1.710)	<.001	1.352 (1.028‐1.780)	.031	1.061 (0.616‐1.826)	.286
Gender		<.001		.208		.038		.947
Female	1		1		1		1	
Male	1.182 (1.137‐1.228)	<.001	1.050 (0.973‐1.132)	.208	1.229 (1.011‐1.494)	.038	0.988 (0.693‐1.409)	.947
Site		<.001		<.001		.307		.216
Rectum	1		1		1		1	
Left colon	2.043 (1.914‐2.181)	<.001	1.320 (1.179‐1.478)	<.001	1.192 (0.893‐1.592)	.234	1.516 (0.826‐2.781)	.179
Right colon	1.718 (1.612‐1.831)	<.001	1.007 (0.901‐1.125)	.900	1.027 (0.776‐1.358)	.853	1.672 (0.940‐2.975)	.080
CEA		<.001		<.001		<.001		<.001
Positive	1		1		1		1	
Negative	0.257 (0.246‐0.268)	<.001	0.319 (0.293‐0.347)	<.001	0.284 (0.228‐0.354)	<.001	0.267 (0.175‐0.408)	<.001
Other	0.137 (0.129‐0.145)	<.001	0.168 (0.150‐0.188)	<.001	0.149 (0.110‐0.202)	<.001	0.229 (0.141‐0.369)	<.001
Grade		<.001		<.001		<.001		<.001
I	1		1		1		1	
II	2.239 (2.030‐2.469)	<.001	1.868 (1.555‐2.243)	<.001	1.849 (1.097‐3.117)	.021	1.714 (0.693‐4.243)	.244
III	3.501 (3.155‐3.884)	<.001	2.656 (2.183‐3.232)	<001	4.918 (2.881‐8.397)	<.001	3.406 (1.039‐11.166)	.002
IV	3.949 (3.466‐4.499)	<.001	2.720 (2.106‐3.514)	<.001	5.895 (3.180‐10.931)	<.001	4.298 (1.691‐10.923)	.043
Histological type		<.001		.150		<.001		.504
AD	1		1		1		1	
MAD	1.663 (1.272‐2.176)	<.001	1.452 (0.910‐2.316)	.117	1.036 (0.721‐1.488)	.848	1.180 (0.165‐8.451)	.869
SRCC	1.897 (1.463‐2.460)	<.001	1.328 (0.817‐2.160)	252	4.326 (2.614‐7.158)	<.001	0.725 (0.087‐6.026)	.766
Tumor size		<.001		<001		<.001		<.001
<5	1		1		1		1	
≥5	1.795 (1.727‐1.866)	<.001	1.912 (1.770‐2.066)	<.001	1.864 (1.530‐2.271)	<.001	2.262 (1.560‐3.280)	<.001
LNH		<.001		<.001		.012		.431
<12	1		1		1		1	
≥12	0.902 (0.860‐0.946)	<.001	0.818 (0.749‐0.893)	<.001	0.754 (0.605‐0.940)	.012	0.831 (0.523‐1.318)	.431
T stage		<.001		<.001		<.001		<.001
T1	1		1		1		1	
T2	1.637 (1.394‐1.922)	<.001	1.141 (0.837‐1.557)	.404	1.058 (0.504‐2.221)	.881	1.588 (0.448‐5.627)	.474
T3	9.023 (7.946‐10.246)	<.001	6.181 (4.914‐7.774)	<.001	4.152 (2.412‐7.147)	<.001	4.992 (1.818‐13.702)	<.001
T4	20.870 (18.342‐23.747)	<.001	15.019 (11.909‐18.942)	<.001	14.582 (8.475‐25.088)	<.001	12.225 (4.404‐33.935)	<.001
N stage		<.001		<.001		<.001		<.001
N0	1		1		1		1	
N1	5.221 (4.947‐5.511)	<.001	4.611 (4.147‐5.127)	<.001	3.868 (2.894‐5.171)	<.001	2.761 (1.693‐4.503)	<.001
N2	11.952 (11.326‐12.613)	<.001	9.130 (8.225‐10.136)	<.001	11.454 (8.767‐14.964)	<.001	7.488 (4.788‐11.713)	<.001

Abbreviations: HR, hazard ratio; CI, confidence interval; CEA, carcinoembryonic antigen; AD, adenocarcinoma; MAD, mucinous adenocarcinoma; SRCC, signet‐ring cell carcinoma; LNH, lymph node harvested.

**TABLE 3:** Multivariate logistic regression for the presence of different metastatic sites at diagnosis of colorectal cancer

	Liver metastasis		Lung metastasis		Bone metastasis		Brain metastasis	
Characteristics	HR (95% CI)	*P*‐value	HR (95% CI)	*P*‐value	HR (95% CI)	*P*‐value	HR (95% CI)	*P*‐value
Age at diagnosis		<.001		.005		.028		.092
<60	1		1		1		1	
≥60	0.701 (0.671‐0.732)	<.001	0.891 (0.821‐0.966)	.005	0.801 (0.657‐0.977)	.028	0.733 (0.511‐1.052)	.092
Race		.245		<.001		−		−
White	1		1					
Black	0.981 (0.951‐1.013)	.400	1.131 (0.999‐1.281)	.052				
Other	1.242 (0.901‐1.318)	.071	1.309 (1.175‐1.458)	<.001				
Gender		<.001		−		.018		−
Female	1				1			
Male	1.259 (1.207‐1.313)	<.001			1.268 (1.042‐1.545)	.018		
Site		<.001		<.001		−		−
Rectum	1		1					
Left colon	2.068 (1.926‐2.219)	<.001	1.203 (1.070‐1.352)	.002				
Right colon	1.837 (1.711‐1.973)	<.001	0.924 (0.820‐1.041)	.193				
CEA		<.001		<.001		<.001		<.001
Positive	1		1		1		1	
Negative	0.376 (0.359‐0.394)	<.001	0.500 (0.458‐0.546)	<.001	0.444 (0.354‐0.557)	<.001	0.405 (0.263‐0.624)	<.001
Other	0.199 (0.188‐0.212)	<.001	0.276 (0.246‐0.310)	<.001	0.249 (0.183‐0.338)	<.001	0.353 (0.216‐0.575)	<.001
Grade		<.001		.002		.002		.222
I	1		1		1		1	
II	1.331 (1.189‐1.490)	<.001	1.008 (0.759‐1.287)	.930	1.223 (0.721‐2.072)	.455	1.162 (0.467‐2.894)	.747
III	1.405 (1.265‐1.560)	<.001	1.042 (0.850‐1.276)	.694	1.804 (1.043‐3.120)	.035	1.275 (0.382‐4.256)	.241
IV	1.412 (1.225‐1.628)	<.001	1.214 (1.007‐1.463)	.042	1.961 (1.041‐3.693)	.037	1.766 (0.682‐4.571)	.692
Histological type		<.001		−		.187		−
AD	1				1			
MAD	3.375 (2.552‐4.463)	<.001			0.807 (0.477‐1.365)	.425		
SRCC	5.621 (4.290‐7.365)	<.001			0.600 (0.323‐1.114)	.106		
Tumor size		<.001		<.001		.126		.034
<5	1		1		1		1	
≥5	1.250 (1.198‐1.305)	<.001	1.337 (1.246‐1.449)	<.001	1.173 (0.956‐1.438)	.126	1.511 (1.032‐2.214)	.034
LNH		<.001		<.001		<.001		−
<12	1		1		1			
≥12	0.610 (0.577‐0.644)	<.001	0.536 (0.488‐0.589)	<.001	0.483 (0.384‐0.606)	<.001		
T stage		<.001		<.001		<.001		.032
T1	1		1		1		1	
T2	1.360 (1.154‐1.602)	<.001	1.077 (0.786‐1.475)	.644	1.002 (0.467‐2.094)	.976	1.314 (0.368‐4.695)	.674
T3	3.639 (3.184‐4.158)	<.001	3.029 (2.380‐3.854)	<.001	1.877 (1.052‐3.348)	.033	2.068 (0.723‐5.917)	.176
T4	5.589 (4.872‐6.412)	<.001	5.052(3.943‐6.472)	<.001	3.998 (2.213‐7.223)	<.001	3.224 (1.087‐9.559)	.035
N stage		<.001		<.001		<.001		<.001
N0	1		1		1		1	
N1	3.489 (3.296‐3.694)	<.001	3.045 (2.726‐3.402)	<.001	2.490 (1.835‐3.378)	<.001	1.818 (1.094‐3.020)	.021
N2	6.933 (6.535‐7.355)	<.001	5.294 (4.724‐5.932)	<.001	5.671 (4.209‐7.671)	<.001	3.709 (2.266‐6.069)	<.001

Abbreviations: HR, hazard ratio; CI, confidence interval; CEA, carcinoembryonic antigen; AD, adenocarcinoma; MAD, mucinous adenocarcinoma; SRCC, signet‐ring cell carcinoma; LNH, lymph node harvested.

**FIGURE 3:**
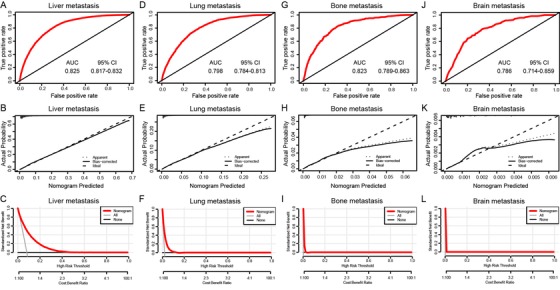
AUC values of ROC predicted liver (A), lung (D), bone (G), and brain (J) metastasis rates of Nomogram. The calibration curve of predictive nomograms for predicting CRC patients’ liver (B), lung (E), bone (H), and brain (K) metastasis rates. Decision curve analysis of the predictive nomogram for predicting liver (C), lung (F), bone (I), and brain (L) metastasis

The ROC curves and the C‐index values were used to appraise the discrimination abilities of the nomograms. The C‐index for the prediction of liver, lung, bone, and brain metastases were 0.82 (95% confidence interval (CI), 0.81‐0.83), 0.80 (95% CI, 0.78‐0.81), 0.83 (95% CI, 0.79‐0.86), and 0.73 (95% CI, 0.72‐0.84), respectively. To ensure that the nomogram forecast models had advantageous efficacy in predicting the specific metastatic sites of CRC patients, logistic ROC analyses were conducted. The area under the curve (AUC) of the nomograms for the prediction of liver, lung, bone, and brain metastases was 0.825 (95% CI, 0.817‐0.832), 0.798 (95% CI, 0.784‐0.813), 0.823 (95% CI, 0.789‐0.863), and 0.786 (95% CI, 0.714‐0.859), respectively (Figure 3A, D, G, and J). In addition, calibration curves of the nomograms used to predict liver, lung, bone, and brain metastases showed no significant deviation from the reference line, which indicated a good degree of confidence (Figure 3B, E, H, and K). DCA is a novel method for appraising alternative prognostic instruments, which takes virtue over the AUC. The DCA curves for the predictive nomogram are presented in Figure 3C, F, I, and L. DCA showed that the predictive nomogram had high net benefits, meaning that it had good clinical implementation significance in predictive specific metastatic sites.

### 3.3 Establishment of a prognostic nomogram integrating clinicopathological features and specific distant metastatic sites

**FIGURE 4:**
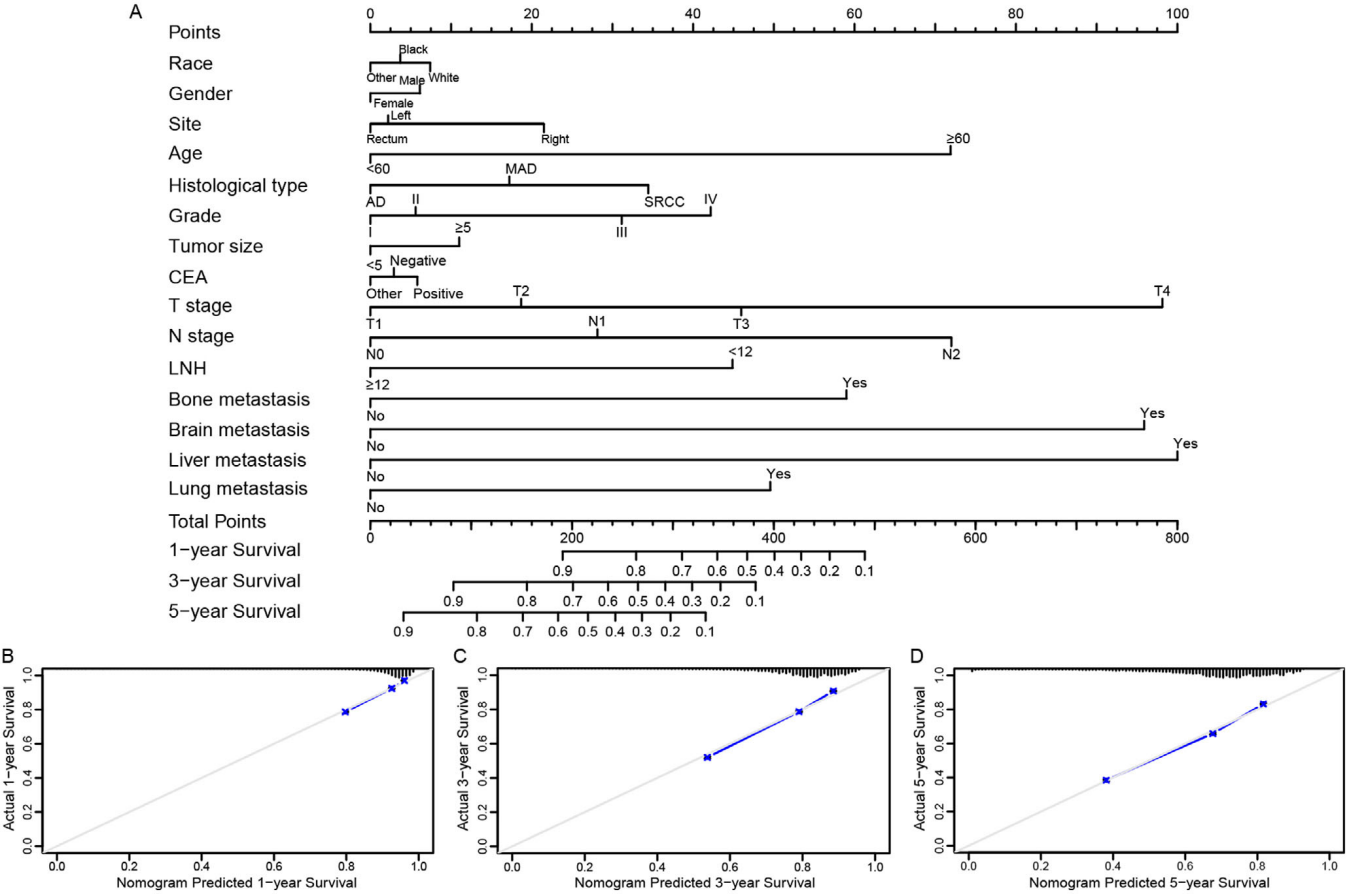
(A) Nomogram convey the results of prognostic models using clinicopathological characteristics and specific metastasis sites to predict overall survival of CRC patients. The calibration curve of prognostic nomogram for predicting patients’ overall survival at 1‐year (B), 3‐year (C), and 5‐year (D)

According to the univariate and multivariate Cox regression analyses results for the OS of CRC patients, all of the significant variables, including race, sex, tumor site, CEA level, grade, age, histological type, tumor size, N stage, LNH, N stage, liver metastases, lung metastases, bone metastases and brain metastases, were integrated to establish the prognostic nomogram for OS (Table 4). The prognostic nomogram for 1‐, 3‐, and 5‐year OS is shown in Figure 4A. By aggregating the scores of each variable and casting the total score on the bottom scale, probabilities can be assessed for 1‐, 3‐, and 5‐year OS. In addition, calibration curves for the nomogram showed no deviations from the reference line, which indicated a high degree of credibility (Figure 4B‐D).

**TABLE 4:** Univariate and multivariate Cox regression for overall survival based on different metastatic sites of colorectal cancer

	Univariate analyses		Multivariate analyses	
Characteristics	HR (95% CI)	*P*‐value	HR (95% CI)	*P*‐value
Age at diagnosis		<.001		<.001
<60	1		1	
≥60	1.847 (1.806‐1.890)	<.001	2.079 (2.031‐2.129)	<.001
Race		<.001		<.001
White	1		1	
Black	0.802 (0.774‐0.831)	<.001	0.826 (0.797‐0.856)	<.001
Other	0.712 (0.663‐0.743)	<.001	0.701 (0.612‐0.721)	<.001
Gender		<.001		<.001
Female	1		1	
Male	1.035 (1.016‐1.055)	<.001	1.078 (1.058‐1.099)	<.001
Site		<.001		<.001
Rectum	1		1	
Left colon	1.259 (1.243‐1.276)	<.001	1.014 (0.983‐1.046)	.380
Right colon	1.312 (1.266‐1.341)	<.001	1.233 (1.196‐1.270)	<.001
CEA		<.001		<.001
Positive	1		1	
Negative	0.626 (0.613‐0.640)	<.001	0.907 (0.887‐0.928)	<.001
Other	0.431 (0.420‐0.442)	<.001	0.667 (0.650‐0.685)	<.001
Grade		<.001		<.001
I	1		1	
II	1.299 (1.249‐1.351)	<.001	1.068 (1.027‐1.112)	.001
III	2.305 (2.209‐2.406)	<.001	1.396 (1.335‐1.459)	<.001
IV	2.738 (2.585‐2.901)	<.001	1.551 (1.462‐1.646)	<.001
Histological type		<.001		<.001
AD	1		1	
MAD	1.409 (1.365‐1.455)	<.001	1.149 (1.113‐1.187)	<.001
SRCC	2.669 (2.484‐2.867)	<.001	1.500 (1.394‐1.615)	<.001
Tumor size		<.001		<.001
<5	1		1	
≥5	1.382 (1.356‐1.408)	<.001	1.098 (1.076‐1.120)	<.001
LNH		<.001		<.001
<12	1		1	
≥12	0.870 (0.851‐0.890)	<.001	0.645 (0.629‐0.660)	<.001
T stage		<.001		<.001
T1	1		1	
T2	1.243 (1.190‐1.299)	<.001	1.234 (1.179‐1.290)	<.001
T3	2.119 (2.046‐2.193)	<.001	1.600 (1.540‐1.661)	<.001
T4	4.719 (4.548‐4.897)	<.001	2.685 (2.576‐2.800)	<.001
N stage		<.001		<.001
N0	1		1	
N1	1.671 (1.634‐1.709)	<.001	1.327 (1.296‐1.360)	<.001
N2	3.278 (3.203‐3.355)	<.001	2.059 (2.005‐2.115)	<.001
Liver metastasis		<.001		<.001
No	1		1	
Yes	4.347 (4.243‐4.454)	<.001	2.589 (2.517‐2.663)	<.001
Lung metastasis		<.001		<.001
No	1		1	
Yes	4.384 (4.192‐4.584)	<.001	1.618 (1.542‐1.698)	<.001
Bone metastasis		<.001		<.001
No	1		1	
Yes	6.461 (5.816‐7.178)	<.001	1.761 (1.581‐1.963)	<.001
Brain metastasis		<.001		<.001
No	1		1	
Yes	7.120 (5.867‐8.639)	<.001	2.460 (2.023‐2.992)	<.001

Abbreviations: HR, hazard ratio; CI, confidence interval; CEA, carcinoembryonic antigen; AD, adenocarcinoma; MAD, mucinous adenocarcinoma; SRCC, signet‐ring cell carcinoma; LNH, lymph node harvested.

**FIGURE 5:**
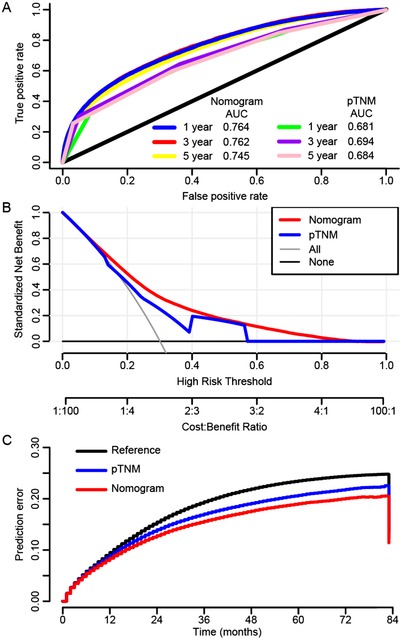
(A) AUC values of ROC predicted 1‐year, 3‐year, and 5‐year overall survival rates of prognostic nomogram and TNM stage. (B) Decision curve analysis of the prognostic nomogram and TNM stage for the overall survival prediction of CRC patients. (C) Prediction error curves for prognostic nomogram and TNM stage to predict patients’ overall survival. Lower prediction errors indicate higher model accuracy

For the prognostic prediction nomogram, the C‐index values and ROC curves were used to evaluate the discrimination power of the nomogram. The C‐index for the prediction of OS was 0.729 (95% CI, 0.724‐0.734). To confirm that the nomogram prediction model had superior efficacy over the TNM staging system in predicting the prognosis of CRC patients, time‐dependent ROC analyses at 1, 3, and 5 years were carried out. The 1‐, 3‐, and 5‐year AUCs were 0.764 (95% CI, 0.741‐0.783), 0.762 (95% CI, 0.745‐0.781), and 0.745 (95% CI, 0.730‐0.761), respectively, for the OS nomogram compared with 0.681 (95% CI, 0.667‐0.694), 0.694 (95% CI, 0.682‐0.705), and 0.684 (95% CI, 0.670‐0.698) for the TNM staging system (Figure 5A).

The DCA curves for the prognostic nomogram and TNM staging system are presented in Figure 5B. Compared with the TNM staging system, DCA showed that the prognostic nomogram had higher net benefits, meaning that it had better clinical implementation significance. The corresponding prediction error curves of the models in Figure 5C showed that the prognostic nomogram had a lower error rate than the TNM staging system, indicating that the nomogram had more accurate discrimination than the TNM staging system.

### 3.4 Prognostic nomogram for risk stratification

**FIGURE 6:**
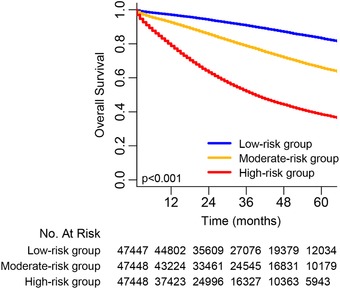
Overall survival in the subgroups according to a tertile of the total score

According to the total score calculated by the prognostic nomogram, all cases were divided into three subgroups, each of which represented a different outcome. The prognosis of each subgroup was reflected by Kaplan‐Meier survival curve, which is shown in Figure 6. Based on OS events, group 1 (low‐risk group) had the highest 5‐year OS of 83.6%, followed by group 2 (moderate‐risk group), with a 5‐year OS of 66.4%; group 3 (high‐risk group) showed the lowest 5‐year OS of 38.8%. Statistically significant distinctions in survival outcomes were noticed among the three groups.

## 4 DISCUSSION

In the current study, nomograms merging clinical and pathological parameters with metastatic information were built to evaluate distant metastasis rates and the 1‐, 3‐, and 5‐year OS probabilities of CRC patients. The identification and calibration of the nomograms were confirmed, and these nomograms have a wide range of applications. According to the ROC, DCA, and error curves, the prognostic nomogram showed better prediction accuracy for CRC than the current TNM staging system. Moreover, the nomogram was qualified to divide patients with CRC into low‐, moderate‐, and high‐risk groups, which suggested that this nomogram could be routinely used for predicting the prognosis of CRC patients.

At present, the diagnosis of young CRC patients has increased. Previous research has demonstrated that age is an independent prognostic factor of CRC patients, with younger age associated with more promising outcomes.^15^ Metastatic prediction nomograms have indicated that CRC patients younger than 60 years were more apt to experience a higher risk of lung, liver, and bone metastases.^16^, ^17^ Otherwise, race (for example, white patients) was related to liver metastasis risk. Furthermore, studies have found that CEA is a prognostic factor and an ideal biomarker for CRC patients.^18^, ^19^, ^20^ Conventional CEA monitoring during the postoperative follow‐up was introduced to monitor relapse and distant metastases after CRC resection surgery. As nomograms manifested, CRC patients with positive CEA levels tended to have significantly worse OS rates and higher metastatic probabilities. In addition, left and right CRCs were indicated to have different embryological origins.^21^ They have miscellaneous features, such as anatomical structure, morphological characteristics, function, and histochemical reactions. A former study associated malignant tumor location with CRC patient prognosis.^21^ Patients with left CRC had a notably higher rate of lung and liver metastases but better prognosis than those with right CRC in view of OS, which was also supposed by this research. Parallel results showed that tumor size was an independent factor for OS in patients with colorectal adenocarcinoma of the ulcerative and infiltrative type in a previous study.^22^ This study proved that larger tumors led to higher risks of lung, liver, and brain metastases, which triggered a worse prognosis.

In addition to age, CEA level, tumor site, and tumor size, preceding studies have also shown that histological differentiation, grade, LNH, N stage, and T stage were independent risk factors for CRC patients.^10^ Histological differentiation was defined as a significant trait to evaluate the advantage of adjuvant chemotherapy in relevant research.^23^ This nomogram verified that low histological differentiation, such as signet ring cell carcinoma (SRCC), was correlated with a worse prognosis. Low histological grade was deliberated among the unfavorable histopathological factors connected with the adverse clinical course of CRC. The results of this investigation showed that high histological grade was strongly suspected to give rise to lung, liver, and bone metastases, while only lung metastases appeared to maintain an association with SRCC. Moreover, NCCN guidelines recommended that the adequate staging of CRC demands at least 12 lymph nodes to be sampled. Previous research inferred that CRC patients with LNH less than 12 tended to have a shorter OS than those with LNH more than 12,^24^ corroborating the results of the nomograms, which indicated that patients with few LNH tended to have a higher risk of lung, liver, and bone metastases. Some scholars suggested that patients with higher T and N stages suffered from a higher risk of liver metastases.^13^ Higher T stage was associated with deeper infiltration, which might result in malignant tumor cells transferring into vessels. The nomograms developed in this study revealed that higher T and N stages were related to a higher risk of lung, liver, bone, and brain metastases and worse survival outcomes.

In this field, much work on the prognostic factors and metastatic sites of CRC has been reported recently. A few researchers reported that their nomogram scoring systems had exceptional capabilities in predicting the prognosis of CRC patients. Previous studies of prognostic prediction in CRC patients have been carried out. For instance, a combination of clinical risk factors and radiomics features emphasized potential advantages to the individualized preoperative prediction of lymph node metastasis in CRC patients, which was proposed to benefit patient OS. Sun et al argued that the fibrinogen and neutrophil‐to‐lymphocyte ratio (F‐NLR score) is a promising predictor for disease relapse in rectal cancer patients.^25^, ^26^ The dissertations mentioned above were dedicated to predicting the preoperative or postoperative conditions of patients, and both might improve the prognosis of patients. However, quite a few studies have proposed examination methods that have a greater trauma or economic cost to patients. Other studies have not considered metastasis in combination with clinical information or could not predict metastases, which greatly impacts CRC patients’ survival outcomes.

However, there are still some shortcomings in the present study. First, therapy information except for surgery, such as specific radiotherapy and chemotherapy therapeutics, was not available in the SEER database to be included into the analysis. Second, the SEER cohort lacks some factors such as detailed mode of presentation and major prognostic scores, which have been demonstrated to have prognostic ability. Third, the SEER database lacks 90% of biomarker expression states, such as KRAS, NRAS, and BRAF. Additional prospective data collection and the internalization of some other variables are encouraged to improve this model.

## 5 CONCLUSIONS

In summary, we developed new nomograms to predict the specific distant metastatic sites and OS probability of CRC patients. The simple and clear nomograms not only have good clinical application value, but also have enough discrimination and calibration ability, which could be used as a convenient tool for clinicians to evaluate the prognosis of individualized CRC patients and determine the treatment strategy.

## ETHICS APPROVAL AND CONSENT TO PARTICIPATE

The Ethical Committee and Institutional Review Board of the Fudan University Shanghai Cancer Center reviewed and approved this study protocol.

## AVAILABILITY OF DATA AND MATERIALS

The dataset used during the study are available from the corresponding author on a reasonable request.

## CONFLICT OF INTEREST

The authors declare no conflict of interest.

## FUNDING INFORMATION

National Natural Science Foundation of China (31470826, 31670858, 81672374)

Science and Technology Commission of Shanghai Municipality (16411966300)

Wu Jieping Medical Foundation of China (320.6750.18136)

## AUTHOR CONTRIBUTIONS

SBM and ZZ had the idea for this study. YQL and XH supervised the acquisition of the data. SBM, XC, and ZZ undertook the statistical analysis. SJC, LZ, and XJM provided statistical advice. All authors contributed to interpretation of the results. SBM, XC, ZZ, and JJP wrote the article and other authors contributed to the content. All authors approved the final version of the manuscript, including the authorship list.
